# Differential cardiovascular benefits of SGLT2 inhibitors, sacubitril/valsartan, omecamtiv mecarbil, and vericiguat across heart failure phenotypes: a systematic review and meta-analysis

**DOI:** 10.3389/fphar.2026.1644757

**Published:** 2026-02-06

**Authors:** Meng Wang, Zhihong Zuo, Ting Wu, Zijing Zhou

**Affiliations:** 1 Department of Pediatrics, West China Second University Hospital, Sichuan University/Key Laboratory of Birth Defects and Related Diseases of Women and Children (Sichuan University), Ministry of Education, Chengdu, China; 2 Department of Critical Care Medicine, Xiangya Hospital, Central South University, Changsha, Hunan, China; 3 Department of Cardiovascular Medicine, National Clinical Research Center for Geriatric Disorders, Xiangya Hospital, Central South University, Changsha, Hunan, China

**Keywords:** heart failure, omecamtiv mecarbil, sacubitril/valsartan, sodium glucose cotransporter-2 inhibitor, vericiguat

## Abstract

**Aim:**

The study evaluated the cardiovascular outcomes associated with pharmacological treatments in heart failure (HF) patients and explored whether the benefits/risks associated with these drugs for HF with mildly reduced or preserved ejection fraction (HFmrEF/HFpEF) were consistent with HF with reduced EF (HFrEF).

**Methods:**

Several online databases were searched. All studies explored the cardiovascular effects of sodium glucose cotransporter-2 inhibitor (SGLT2i), sacubitril/valsartan, omecamtiv mecarbil and vericiguat were screened and reviewed.

**Results:**

A total of 39 studies were included. Compared with placebo therapy, SGLT2i significantly reduced cardiovascular death and hospitalization for HF (HHF) in both HFrEF and HFmrEF/HFpEF patients (approximately 13%–27% risk reduction). SGLT2i reduced serious adverse events across all HF types. Sacubitril/valsartan demonstrated significant benefits in HFrEF patients, reducing cardiovascular death by 19%, all-cause mortality by 22%, and HHF by 22%. However, these benefits were not observed in HFmrEF/HFpEF patients. In contrast, sacubitril/valsartan substantially increased hypotension risk in HFmrEF/HFpEF patients. Omecamtiv mecarbil and vericiguat tended to improve cardiovascular outcomes in patients with HF, but the difference was not statistically significant.

**Conclusion:**

SGLT2i represents an effective and safe treatment strategy across the HF spectrum. Sacubitril/valsartan significantly improves outcomes in HFrEF but requires careful benefit-risk evaluation in HFmrEF/HFpEF patients. Current evidence does not support routine use of omecamtiv mecarbil or vericiguat. Large-scale randomized trials are warranted to validate these findings.

**Systematic review registration:**

CRD42023455966.

## Introduction

Approximately 64 million people worldwide have heart failure (HF). In developed countries, the overall prevalence of HF is elevated and increases with age. Based on the left ventricular ejection fraction (LVEF), HF is generally divided into HF with reduced ejection fraction (EF) defined as LVEF ≤40%, HF with mildly reduced EF (HFmrEF) with LVEF between 41% and 49%, and HF with preserved EF (HFpEF) defined as LVEF≥50% (Cleland et al., 2018; Lund et al., 2018; Tsutsui, 2022). Despite therapeutic advances, prognosis remains poor across the HF spectrum, with persistently high mortality rates and progressive symptom burden, underscoring the urgent need for more effective treatment strategies.

Recent landmark clinical trials have demonstrated cardiovascular benefits of novel pharmacological agents, including sodium-glucose cotransporter-2 inhibitors (SGLT2i) (Packer et al., 2020), sacubitril/valsartan (McMurray et al., 2014), omecamtiv mecarbil (Teerlink et al., 2021), and vericiguat (Armstrong et al., 2020a). While substantial progress has been achieved in HFrEF management, therapeutic development for HFmrEF/HFpEF has lagged considerably. Emerging evidence suggests that certain HFrEF therapies may extend benefits to patients with HFmrEF/HFpEF (Anker et al., 2021; Vaduganathan et al., 2023); however, direct comparative evidence across the HF spectrum remains limited, and the benefit-risk profiles of these agents in different HF phenotypes have not been systematically synthesized.

Therefore, this meta-analysis aims to synthesize contemporary evidence to directly compare the efficacy and safety profiles of SGLT2i, sacubitril/valsartan, omecamtiv mecarbil, and vericiguat, and firstly to evaluate whether their benefit-risk ratios are consistent across patients with HFrEF and HFmrEF/HFpEF. This comprehensive synthesis will inform evidence-based treatment decisions and guide future therapeutic development for the diverse HF population.

## Methods

This study was registered in PROSPERO, with registration No. CRD42023455966.

### Search strategy

A comprehensive systematic literature search was conducted in five electronic databases: Web of Science, PubMed, the Cochrane Library, EMBASE, and China National Knowledge Infrastructure (CNKI), from inception to 17 February 2025, without language restrictions. The complete search strategies for all databases are provided in Supplementary Material.

### Inclusion and exclusion criteria

The following inclusion criteria were applied: (1) type of participants: patients (≥18 years old) in each study who were diagnosed with HF (we defined HFrEF as LVEF ≤40% and HFmrEF/HFpEF >40%) and (2) type of study: clinical studies that provide information about the effectiveness and safety of the anti-HF agents mentioned above. Exclusion criteria included (1) study design: reviews, comments, letters, case reports, and abstracts; (2) type of participants: animals, patients<18 years old, and pregnant women; and (3) insufficient information concerning evaluation rates.

### Outcomes

The primary outcomes evaluated in this study were cardiovascular death, hospitalization for HF (HHF), and death from any cause. The second outcome included serious adverse events, adverse events of special interest, the change in the level (relative to baseline) of N-Terminal Pro-Brain Natriuretic Peptide (NT-ProBNP), systolic blood pressure (SBP), and EF.

### Study selection

After removing duplicates, the remaining identified trials were reviewed by two independent investigators to confirm that they fulfilled the inclusion criteria. The reference lists of included studies were screened and assessed in the same manner. When discrepancies occurred, all authors rechecked the data source. The final decision was made based on the agreement of all authors.

### Data extraction

The extracted data included the last name of the first author, year of publication, sample size (N), mean age (years), drug names, primary outcomes, HF type, and research type. The Newcastle-Ottawa scale (NOS) was employed for the quality assessment of the included retrospective studies. Score of 1–3, 4–6, and 7–9 presented low, intermediate, and high quality, respectively. For randomized controlled trials (RCTs), we used the Cochrane risk-of-bias tool to evaluate their quality. Seven domains (randomization, allocation concealment, blinding of participants, incomplete data, selective reporting, other bias) were rated as low, high, or unclear risk based on Cochrane criteria. Overall risk of bias for each trial was determined conservatively: trials with all seven domains rated as low risk were classified as low overall risk; trials with any high-risk domain or more than two unclear-risk domains were classified as high overall risk; remaining trials were classified as moderate overall risk. All authors resolved disagreements through discussion.

### Statistical analysis

We employed the weighted mean difference (WMD) and risk ratio (RR) to compare continuous and dichotomous variables, respectively. The 95% confidence interval (CI) is presented in the reports of all results. Random-effect models were used to pool the effect estimates of the outcomes. Egger’s and Begg’s tests (P < 0.10) were conducted to evaluate the possible publication bias of the outcome, supplemented by visual inspection of funnel plot symmetry. Sensitivity analyses were conducted by removing one study at a time to observe the effect estimates of the outcomes. All statistical analyses were performed using STATA statistical software (version 12.0; STATA Corporation, College Station, Texas, United States).

## Results

### Selection of included studies and study characteristics

**FIGURE 1:**
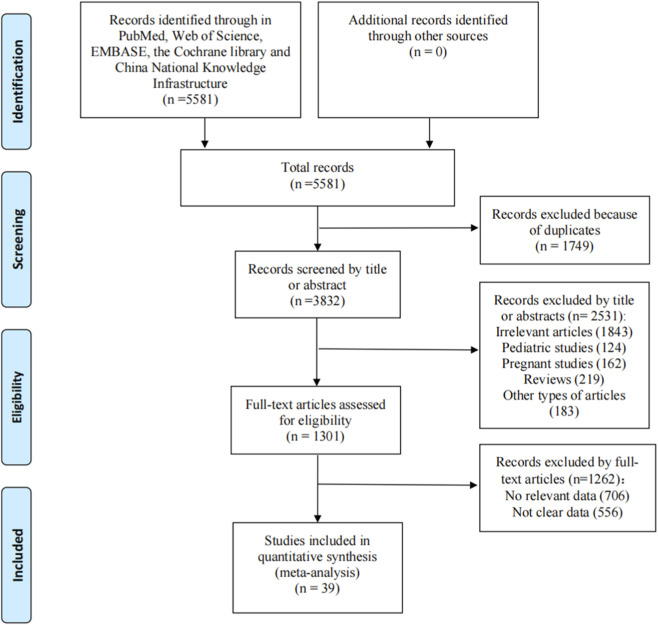
Flowchart depicting a systematic review process.

A total of 5581 relevant articles were identified by searching several online databases. Figure 1 presents the review and selection process for eligible trials in this study. Finally, 39 studies (Packer et al., 2020; Teerlink et al., 2021; Armstrong et al., 2020a; Anker et al., 2021; Vaduganathan et al., 2023; McMurray et al., 2019; Nassif et al., 2019; Singh et al., 2020; Nassif et al., 2021; Solomon et al., 2022; Savarese et al., 2021; Pietschner et al., 2021; Mordi et al., 2020; Omar et al., 2021; Polito et al., 2020; Greene et al., 2021; Chen et al., 2021; Rattanavipanon et al., 2021; Mirić et al., 2021; Gao et al., 2020; Solomon et al., 2019; Hsieh et al., 2021; Chang et al., 2020; He et al., 2021; Damman et al., 2018; Li et al., 2021; Solomon et al., 2012; Velazquez et al., 2019; Piepoli et al., 2021; Riaz et al., 2021; Lewis et al., 2022; Teerlink et al., 2016a; Teerlink et al., 2016b; Gheorghiade et al., 2015; Armstrong et al., 2020b; Pieske et al., 2021; Mentz et al., 2023; Kosiborod et al., 2017; Si, 2023; Dong and Yuan, 2023) were included in the meta-analysis, and their characteristics are summarized in Supplementary Table S1. Among these studies, 34 were RCTs and 5 were retrospective studies. Eleven trials focused on the effects of SGLT2i in patients with HF, while 21 evaluated the efficiency of sacubitril/valsartan. Omecamtiv mecarbil was discussed in 4 articles, while the cardiovascular outcomes of vericiguat were presented in 3 trials. Totally, 53952 patients with HFrEF were included in the study, and 33737 patients with HFpEF were included. Of the 34 RCTs included, 22 (64.7%) demonstrated low risk of bias across all domains, 5 (14.7%) had moderate risk, and 7 (20.6%) had high risk of bias, primarily related to inadequate randomization or blinding. All retrospective studies were rated as high quality (see Supplementary Tables S1, S2).

### SGLT2i

**FIGURE 2:**
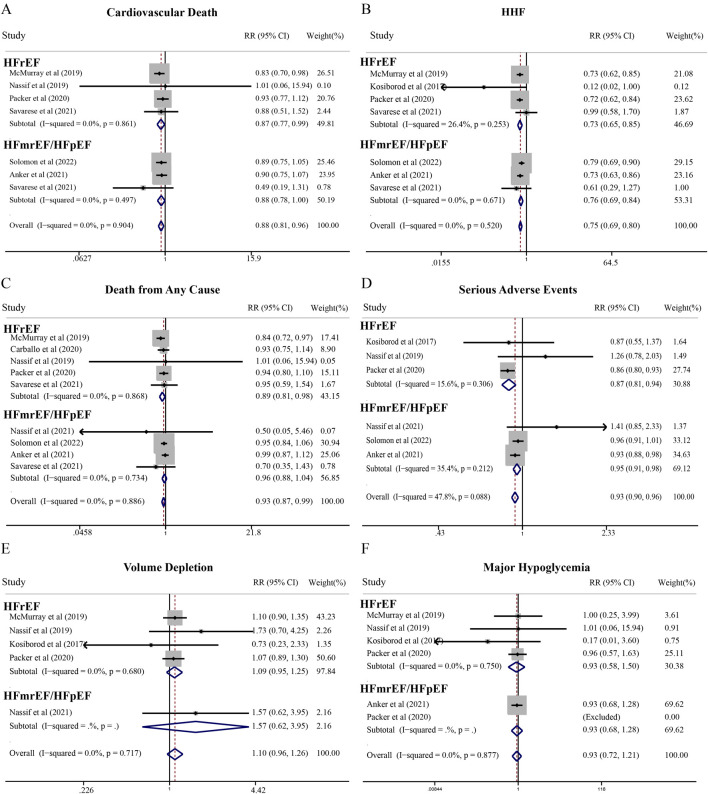
Meta-analysis of cardiovascular effects and adverse events of SGLT2i compared with standard treatment therapy in patients with HF. **(A)** Cardiovascular death, **(B)** Hospitalization for heart failure (HHF), **(C)** Death from any cause, **(D)** Serious adverse events, **(E)** Volume depletion, and **(F)** Major hypoglycemia.

Compared with placebo, SGLT2i significantly reduced the risk of cardiovascular death in patients with HF (RR = 0.88; 95% CI, 0.81–0.96). Subgroup analyses demonstrated consistent benefits across HF phenotypes: HFrEF (RR = 0.87; 95% CI, 0.77–0.99) and HFmrEF/HFpEF (RR = 0.88; 95% CI, 0.78–1.00) (Figure 2). For all-cause mortality, SGLT2i significantly reduced risk in HFrEF patients (RR = 0.89; 95% CI, 0.81–0.98), while showing a non-significant trend in HFmrEF/HFpEF patients (RR = 0.96; 95% CI, 0.88–1.04). Importantly, SGLT2i significantly decreased HHF risk across the entire HF spectrum (RR = 0.75; 95% CI, 0.70–0.80) (Figure 2). Notably, the SGLT2i cardiovascular death benefit in HFmrEF/HFpEF demonstrated borderline statistical significance. However, the point estimate indicates a clinically meaningful 12% risk reduction, and is corroborated by highly significant HHF reduction in the same population. Nevertheless, additional evidence may be needed to definitively establish mortality benefits in HFmrEF/HFpEF patients.

**FIGURE 3:**
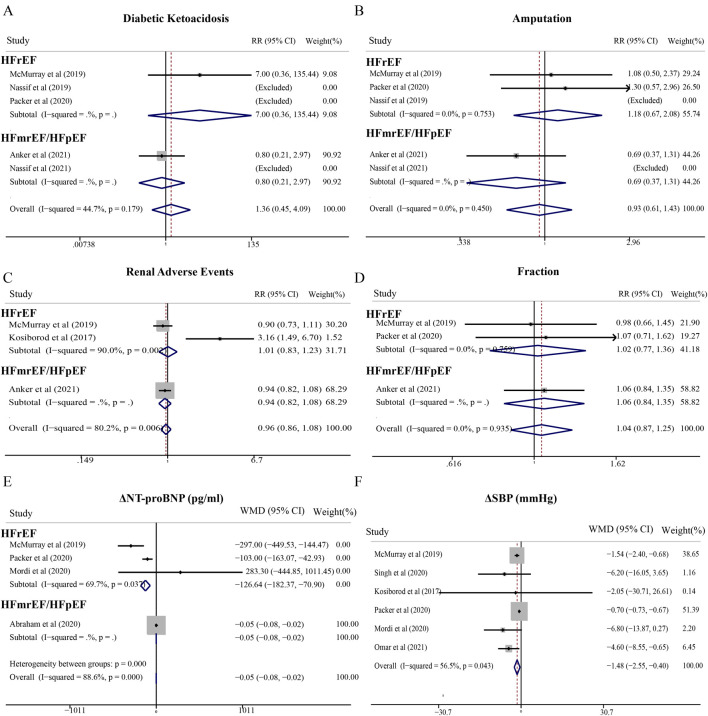
Meta-analysis of secondary outcomes of SGLT2i compared with standard treatment therapy in patients with HF. **(A)** Diabetic ketoacidosis, **(B)** Amputation, **(C)** Renal adverse events, **(D)** Fraction, **(E)** NT-proBNP levels, and **(F)** systolic blood pressure.

Regarding safety, SGLT2i significantly reduced serious adverse events in both HFrEF (RR = 0.87; 95% CI, 0.81–0.94) and HFmrEF/HFpEF (RR = 0.95; 95% CI, 0.91–0.98) patients, without increasing adverse events of interest including volume depletion, major hypoglycemia, or renal adverse events (Figure 2). Additionally, SGLT2i favorably impacted NT-proBNP (WMD = −126.64 pg/mL; 95% CI, −182.37 to −70.90 pg/mL) and systolic blood pressure (WMD = −1.48 mmHg; 95% CI, −2.55 to −0.40 mmHg) in HFrEF patients (Figure 3).

### Sacubitril/valsartan

**FIGURE 4:**
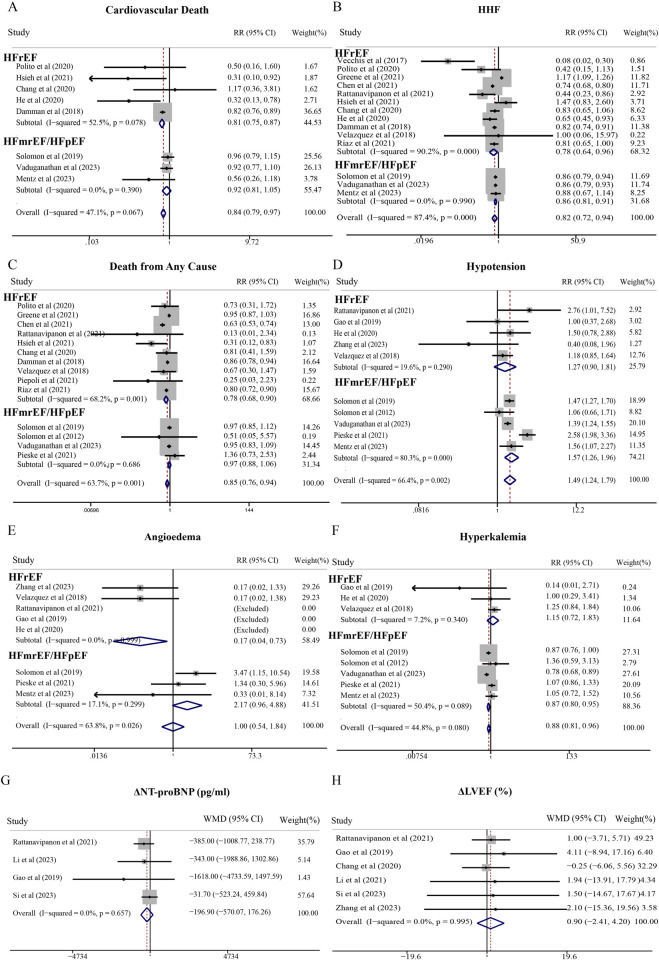
Meta-analysis of cardiovascular effects and adverse events of sacubitril/valsartan compared with standard treatment therapy in patients with HF. **(A)** Cardiovascular death, **(B)** Hospitalization for heart failure (HHF), **(C)** Death from any cause, **(D)** Hypotension, **(E)** Angioedema, **(F)** Hyperkalemia, **(G)** NT-proBNP levels, and **(H)** Ejection fraction levels.

In HFrEF patients, sacubitril/valsartan demonstrated significant reductions in cardiovascular death (RR = 0.81; 95% CI, 0.75–0.87), all-cause mortality (RR = 0.78; 95% CI, 0.68–0.90), and HHF (RR = 0.78; 95% CI, 0.64–0.96) compared with control therapy (Figure 4). Conversely, in HFmrEF/HFpEF patients, sacubitril/valsartan reduced HHF risk (RR = 0.86; 95% CI, 0.81–0.91) but showed no significant effect on cardiovascular death (RR = 0.92; 95% CI, 0.81–1.05) or all-cause mortality (RR = 0.97; 95% CI, 0.88–1.06) (Figure 4). Notably, sacubitril/valsartan substantially increased hypotension risk in HFmrEF/HFpEF patients (RR = 1.57; 95% CI, 1.26–1.96), an effect not observed in HFrEF patients (RR = 1.27; 95% CI, 0.90–1.81). For angioedema, the overall relative risk was 1.00 (95% CI: 0.54–1.84), with a lower risk observed in HFrEF patients (RR 0.17, 95% CI: 0.06–0.73) compared to HFmrEF/HFpEF patients (RR 2.17, 95% CI: 0.96–4.88) (Figure 4E). Regarding hyperkalemia, the pooled analysis demonstrated a modest reduction in risk (RR 0.88, 95% CI: 0.81–0.96), particularly pronounced in the HFmrEF/HFpEF subgroup (RR 0.87, 95% CI: 0.80–0.95) (Figure 4F). Sacubitril/valsartan showed no significant effects on NT-proBNP or LVEF in HFrEF patients (Figures 4G, H).

### Omecamtiv mecarbil

**FIGURE 5:**
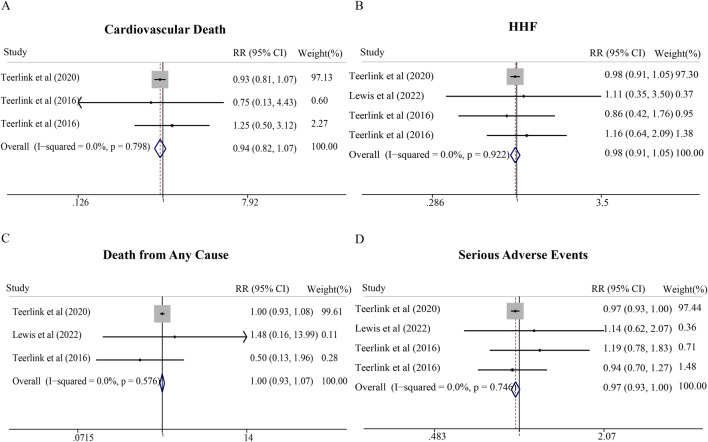
Meta-analysis of cardiovascular effects and serious adverse events of omecamtiv mecarbil compared with standard treatment therapy in patients with HF. **(A)** Cardiovascular death, **(B)** Hospitalization for heart failure (HHF), **(C)** Death from any cause, and **(D)** Serious adverse events.

Four high-quality RCTs enrolling 9,019 HFrEF patients (all rated as low risk of bias) were included in this analysis. Despite trends toward cardiovascular benefit, omecamtiv mecarbil did not significantly reduce cardiovascular death (RR = 0.94; 95% CI, 0.82–1.07), all-cause mortality (RR = 1.00; 95% CI, 0.93–1.07), HHF (RR = 0.98; 95% CI, 0.91–1.05), or serious adverse events (RR = 0.97; 95% CI, 0.93–1.00) compared with placebo (Figure 5). The CI for serious adverse events (0.93–1.00) approached but did not cross unity, suggesting a potential safety benefit that may require larger trials to definitively establish. No studies evaluating omecamtiv mecarbil in HFmrEF/HFpEF populations were identified.

### Vericiguat

**FIGURE 6:**
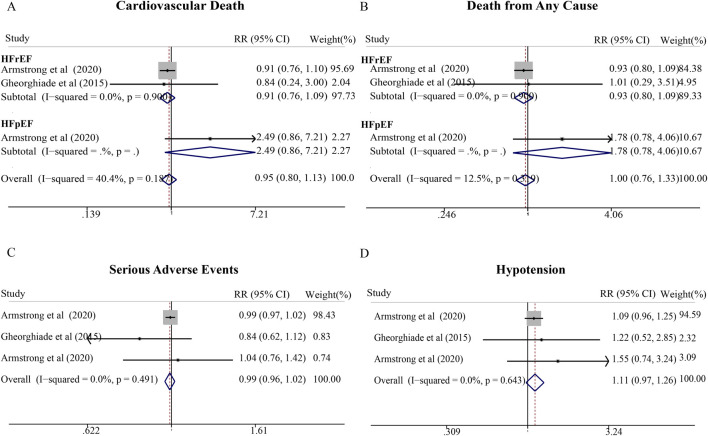
Meta-analysis of cardiovascular effects and hypotension of vericiguat compared with standard treatment therapy in patients with HF. **(A)** Cardiovascular death, **(B)** Hospitalization for heart failure (HHF), **(C)** Serious adverse events, and **(D)** Hypotension.

Three RCTs comprising 5,296 patients (predominantly HFrEF) were analyzed, all demonstrating low risk of bias. Similar to omecamtiv mecarbil, vericiguat showed no significant differences compared with placebo for cardiovascular death (RR = 0.95; 95% CI, 0.80–1.13), all-cause mortality (RR = 1.00; 95% CI, 0.76–1.33), serious adverse events (RR = 0.99; 95% CI, 0.96–1.02), or hypotension (RR = 1.11; 95% CI, 0.97–1.26) (Figure 6). While point estimates suggested modest trends toward benefit for mortality outcomes, the wide confidence intervals and relatively limited number of trials may have constrained statistical power to detect clinically meaningful effects. Further large-scale trials, particularly in HFmrEF/HFpEF populations where data remain scarce, are warranted to clarify the therapeutic role of these agents.

### Publication bias and sensitivity analysis

The Egger’s test result for angioedema (p = 0.066) suggested potential publication bias. Angioedema is a relatively rare adverse event, resulting in a limited number of studies reporting this outcome. Egger’s test has reduced statistical power with fewer than 10 studies, increasing the risk of both false-positive and false-negative results. Therefore, more trials are needed to confirm the conclusion. No significant publication bias and no obvious visual asymmetry was observed in other study (Supplementary Table S3; Supplementary Figures S1, S2). The sensitivity analysis revealed no significant differences in the outcomes.

## Discussion

In this meta-analysis of 39 trials, we systematically evaluated the cardiovascular efficacy and safety of four novel HF pharmacotherapies across the ejection fraction spectrum. Our findings demonstrate that SGLT2i consistently reduced cardiovascular death, all-cause mortality, and HHF in both HFrEF and HFmrEF/HFpEF patients, with favorable effects on systolic blood pressure and NT-proBNP levels without increasing adverse events. Sacubitril/valsartan significantly improved outcomes in HFrEF but showed limited mortality benefit and increased hypotension risk in HFmrEF/HFpEF. Omecamtiv mecarbil and vericiguat demonstrated no significant cardiovascular benefits in the pooled analyses. These divergent findings underscore the importance of phenotype-specific therapeutic responses in contemporary HF management.

A central finding of this meta-analysis is the contrasting efficacy profile between SGLT2i and other novel agents. SGLT2i demonstrated consistent cardiovascular benefits regardless of LVEF, whereas sacubitril/valsartan, omecamtiv mecarbil, and vericiguat exhibited phenotype-dependent or absent efficacy. The contrasting efficacy of treatments in HFrEF versus HFmrEF/HFpEF reflects fundamental pathophysiological differences between these phenotypes (Shah et al., 2012). HFrEF is characterized by systolic dysfunction with eccentric remodeling, cardiomyocyte loss, and prominent neurohormonal activation—particularly marked elevation of RAAS components and compensatory natriuretic peptide release (Hartupee and Mann, 2017; Nauta et al., 2020). In contrast, HFpEF/HFmrEF are heterogeneous syndromes dominated by diastolic dysfunction, concentric remodeling, myocardial stiffness, and microvascular endothelial dysfunction, with less consistent or pronounced neurohormonal activation. The pathophysiology involves multiple mechanisms including cardiomyocyte hypertrophy, interstitial fibrosis, impaired myocardial energetics, chronotropic incompetence, and increased ventricular-arterial stiffness (Roh et al., 2022; Paulus and Zile, 2021; Singh et al., 2019).

### SGLT2i

Recent evidence has reinforced the therapeutic role of SGLT2 inhibitors across the HF spectrum. Extended DELIVER follow-up and EMPEROR-Preserved pooled analyses confirm consistent mortality benefits across all LVEF ranges (Vaduganathan et al., 2022; Ferreira et al., 2024), corroborating our findings of cardiovascular mortality reduction in both HFrEF and HFmrEF/HFpEF populations. Previous study also demonstrated SGLT2i therapy was associated with a reduced risk of cardiovascular death or HHF compared to placebo, vericiguat, and omecamtiv mecarbil among patients with HFrEF (Pagnesi et al., 2022). Aimo et al. (2021) reported SGLT2i demonstrated the greatest effect on HHF over the standard therapy, as well as a significant benefit over vericiguat. The universal efficacy of SGLT2i across the HF spectrum can be attributed to its multifaceted mechanisms that address pathophysiological abnormalities common to all HF phenotypes. SGLT2i improves ventricular loading conditions through osmotic diuresis and blood pressure reduction (Milton et al., 2017; Naveed et al., 2016; Wang et al., 2022), enhances endothelial function via voltage-gated potassium channel activation, optimizes myocardial energetics by promoting fatty acid oxidation, reduces oxidative stress through Na^+^/H+ exchanger inhibition, and favorably modulates cardiovascular risk factors including uric acid (Yuliya et al., 2014), body weight (Mishriky et al., 2018), and lipid profiles (Wang et al., 2022; Bechmann et al., 2024). SGLT2i also demonstrate important pleiotropic cardiovascular actions. A 2025 meta-analysis of 58,569 participants showed that SGLT2i reduce adjudicated sudden cardiac death by 18% (Matteucci et al., 2025), mediated through ventricular remodeling attenuation, anti-fibrotic effects, and improved cardiac energetics (Qu et al., 2025; Philippaert et al., 2021). Importantly, these mechanisms operate independently of neurohormonal activation status or systolic function, explaining the consistent benefit observed in both preserved and reduced EF populations. Our findings reinforce that SGLT2i represents a foundational therapy for the entire HF spectrum, with a favorable benefit-risk profile characterized by significant reductions in serious adverse events across all phenotypes. The 2023 guidelines now provide Class I, Level of Evidence A recommendations for SGLT2 inhibitors in both HFmrEF and HFpEF patients (McDonagh et al., 2023).

### Sacubitril/valsartan

In contrast, sacubitril/valsartan’s mechanism—dual inhibition of angiotensin receptors and neprilysin to promote natriuresis, vasodilation, and reverse remodeling through modulation of RAAS and natriuretic peptide pathways—appears most effective in HFrEF, where neurohormonal activation is prominent (Julio and Nancy, 2017; Hai-Ping et al., 2018; Je et al., 2017). Our meta-analysis confirmed substantial mortality and morbidity reductions in HFrEF patients, consistent with landmark trials. However, the lack of mortality benefit in HFmrEF/HFpEF suggests that these phenotypes may not exhibit the same degree of neurohormonal dysregulation, or that competing pathophysiological mechanisms (such as concentric remodeling, microvascular dysfunction, and myocardial stiffness) predominate and are less responsive to RAAS-neprilysin modulation. A critical safety finding was the substantially elevated hypotension risk with sacubitril/valsartan in HFmrEF/HFpEF patients, contrasting with the non-significant trend in HFrEF. Alberto et al. (2024) demonstrated that higher baseline LVEF was independently associated with increased hypotension risk in sacubitril/valsartan-treated patients, supporting the hypothesis of differential hemodynamic reserve across HF phenotypes. HFmrEF/HFpEF populations in our included trials exhibited demographic and clinical characteristics that predispose to hypotension. HFmrEF/HFpEF populations are characterized by advanced age (mean 72–75 years) (Supplementary Table S1), female predominance (50%–60%) (Lam et al., 2019), higher baseline blood pressure (reflecting chronic hypertension) (Shah et al., 2016), and greater comorbidity burden (CKD 40%–50%, diabetes 45%–50%, atrial fibrillation 40%–45%) (Jafari et al., 2023; Dunlay et al., 2017)—all predisposing factors for hemodynamic intolerance. Many HFpEF patients exhibit impaired heart rate response to physiological stress, limiting their ability to compensate for reduced blood pressure through increased cardiac output (Borlaug et al., 2010). Sacubitril/valsartan holds a Class I, Level of Evidence B recommendation in the ESC guidelines for HFrEF patients (McDonagh et al., 2023). For HFmrEF and HFpEF, the guideline recommendations are more nuanced. The 2021 ESC guidelines provided a Class IIb recommendation for sacubitril/valsartan in HFmrEF. The current ESC position reflects appropriate caution, awaiting dedicated prospective trials specifically designed for the HFmrEF population before upgrading recommendations to Class I.

### Omecamtiv mecarbil

Omecamtiv mecarbil selectively enhances myocardial contractility by increasing cardiac myosin’s pre-powerstroke state without altering calcium handling, thereby avoiding conventional inotrope-related toxicity (Vicente et al., 2017; Fady et al., 2011; John R,T. et al., 2011; John GF,C. et al., 2011). Despite favorable effects on cardiac structure and function in the COSMIC-HF trial (Teerlink et al., 2016a), our pooled analysis revealed no significant impact on cardiovascular death, all-cause mortality, or HHF. The GALACTIC-HF trial demonstrated only a modest reduction in the composite endpoint (HR 0.92, 95% CI 0.86–0.99) without improvements in individual mortality or symptom outcomes, suggesting that enhanced contractility alone may be insufficient to alter hard clinical endpoints in HF management where multidrug optimization is standard (Teerlink et al., 2021).

### Vericiguat

Similarly, vericiguat stimulates soluble guanylate cyclase to enhance the NO-sGC-cGMP pathway, addressing endothelial dysfunction and impaired vasomotor regulation characteristic of HF. However, our analysis found no significant mortality or morbidity benefits. While the VICTORIA trial reported a reduction in the composite outcome of cardiovascular death or first HHF (driven primarily by HHF reduction), individual mortality endpoints remained unchanged, and the VITALITY-HFpEF trial (Armstrong et al., 2020b) showed no symptomatic improvement in HFpEF patients. In our opinion, further clinical trials of vericiguat are needed to determine the potential role of this drug in patients with HF, due to limited and contradictory results. In contrast to the foundational pillars of HF therapy, vericiguat and omecamtiv mecarbil occupy more selective therapeutic niches, reflected in their Class IIb, Level of Evidence B recommendations in the 2021 ESC guidelines for HFrEF (McDonagh et al., 2021). These weaker recommendations stem from their more limited scope of proven benefit and specific target populations.

Beyond the four drug classes included in our meta-analysis, other therapeutic agents, such as mineralocorticoid Receptor Antagonists (MRAs), have established roles in heart failure management (Bismpos et al., 2024). MRAs have demonstrated robust and well-established benefits in HFrEF patients. Current guidelines provide Class I, Level of Evidence A recommendations for MRAs in symptomatic HFrEF patients (McDonagh et al., 2023; Heidenreich et al., 2022). The role of MRAs in HFmrEF and HFpEF has been more controversial, though emerging evidence suggests potential benefits in selected populations. The TOPCAT trial, which evaluated spironolactone in patients with symptomatic HFpEF (LVEF ≥45%), did not demonstrate a significant reduction in the primary composite outcome of cardiovascular death, aborted cardiac arrest, or HHF (Pitt et al., 2014). However, spironolactone did significantly reduce HHF. Importantly, subgroup analyses suggested benefit in patients with elevated natriuretic peptides and in specific geographic regions (Pfeffer et al., 2015). Post-hoc analyses from trials including patients with LVEF in the mildly reduced range have suggested dose-dependent benefits similar to those observed in HFrEF (Straw et al., 2023). Current ESC guidelines provide a Class IIb recommendation for MRA use in HFmrEF patients (McDonagh et al., 2023).

ACE inhibitors represent one of the foundational pillars of HFrEF therapy, with decades of evidence supporting their use. Current guidelines recommend ACE inhibitors as Class I, Level of Evidence A therapy for all patients with HFrEF, unless contraindicated or not tolerated (McDonagh et al., 2023). In contrast to their established benefits in HFrEF, the role of ACE inhibitors in HFpEF has been disappointing. The PEP-CHF trial, which evaluated perindopril in elderly patients (≥70 years) with HFpEF, demonstrated a non-significant trend toward reduction in the primary composite endpoint of all-cause mortality or unplanned HHF (Cleland et al., 2006). Similarly, the CHARM-Preserved trial showed that the angiotensin receptor blocker (ARB) candesartan did not significantly reduce the primary composite endpoint of cardiovascular death or HHF in HFpEF patients, though there was a modest reduction in HHF (Yusuf et al., 2003). However, *post hoc* analyses suggest that ACE inhibitors and ARB may provide benefits in HFmrEF patients, with current guidelines providing Class IIb recommendations for their use in this population (McDonagh et al., 2023; Straw et al., 2023).

### Limitation

This meta-analysis has several limitations. First, the combined analysis of HFmrEF and HFpEF patients may obscure phenotype-specific treatment differences, as separate subgroup data were not consistently available in the included trials. Second, the inclusion of studies from CNKI may introduce geographic and linguistic bias. Clinical practices and patient demographics in Asian populations may differ from Western countries. Sensitivity analyses excluding these studies yielded consistent results. Third, owing to the limited number of clinical trials for omecamtiv mecarbil and vericiguat, it is difficult to evaluate the exact benefits of these novel drugs. However, we summarized recent clinical trials about omecamtiv mecarbil and vericiguat in the discussion, which aimed to provide evidence for clinical physicians to choose drugs. Forth, there was a difference in the duration of follow-up between trials. However, the heterogeneity of the results was very low. Fifth, several findings showed confidence intervals approaching 1.00, warranting cautious interpretation. For example, the SGLT2i cardiovascular death benefit in HFmrEF/HFpEF demonstrated borderline statistical significance. However, the point estimate indicates a clinically meaningful 12% risk reduction, and is corroborated by highly significant HHF reduction in the same population. Nevertheless, additional high-quality evidence is needed to confirm these borderline findings and establish definitive conclusions. Sixth, several studies were rated as high risk. Sensitivity analyses excluding these studies yielded consistent results. Additionally, we attempted meta-regression to explore LVEF as a continuous moderator. However, when stratified by drug class and HF subtype, most subgroups contained <10 studies (Cochrane Handbook), below the recommended minimum for reliable meta-regression. Finally, some of the trials were retrospective studies, retrospective studies are susceptible to selection bias, as treatment allocation was not randomized and may have been influenced by physician preference, disease severity, or patient characteristics not fully captured in multivariable adjustments. Additionally, information bias may arise from incomplete or inconsistent documentation of clinical outcomes, covariates, and adverse events in retrospective medical records, potentially leading to misclassification or underreporting.

## Conclusion

SGLT2i was an effective and safe strategy to improve the cardiovascular benefits in HFrEF and HFmrEF/HFpEF patients. Sacubitril/valsartan treatment significantly improved cardiovascular outcomes in patients with HFrEF. However, benefit/risk ratios of sacubitril/valsartan applying for HFpEF/HFmrEF patients should be carefully evaluated. Current evidence does not support routine use of omecamtiv mecarbil or vericiguat, and more trials should be conducted to obtain validated data.

## Data Availability

The datasets presented in this study can be found in online repositories. The names of the repository/repositories and accession number(s) can be found in the article/Supplementary Material.
