# Supplementary material for: Differential cardiovascular benefits of SGLT2 inhibitors, sacubitril/valsartan, omecamtiv mecarbil, and vericiguat across heart failure phenotypes: a systematic review and meta-analysis
Source: Front Pharmacol. 2026 Feb 6;17:1644757. doi: 10.3389/fphar.2026.1644757 (PMC12920234; doi:10.3389/fphar.2026.1644757)
Supplement: Supplementary file 1 [file Supplementaryfile1.docx]

Supplementary Table 1 Study characteristics of included studies

| Author | Year | Population(n) | | Age(years) | | Drug (%) | | Primary  outcomes | Research  type | Type | Quality | Follow-up Duration |  |
| --- | --- | --- | --- | --- | --- | --- | --- | --- | --- | --- | --- | --- | --- |
|  |  | Intervention | Comparator | Intervention | Comparator | Intervention | Comparator |  |  |  |  |  |  |
| McMurray  et al^10^  (DAPA-HF) | 2019 | 2373 | 2371 | 66.2 | 66.5 | Dapagliflozin | Standard drug therapy | Worsening HF, death from cardiovascular  causes | RCT | HFrEF | NA | 18.2 months |  |
| Nassif et al^11^  (DEFINE-HF) | 2019 | 131 | 132 | 62.2 | 60.4 | Dapagliflozin | Standard drug therapy | HF or urgent HF visits | RCT | HFrEF | NA | 12 weeks |  |
| Kosiborod  et.al^42^ | 2017 | 171 | 149 | 63.6 | 64.9 | Dapagliflozin | Standard drug therapy | Major adverse cardiac events; events of HHF | RCT | HFrEF | NA | 52 weeks |  |
| Packer et al^4^  (EMPEROR-Reduced) | 2020 | 1863 | 1867 | 67.2 | 66.5 | Empagliflozin | Standard drug therapy | Cardiovascular death, HHF | RCT | HFrEF | NA | 16 months |  |
| Singh et al^12^ | 2020 | 28 | 28 | NR | NR | Dapagliflozin | Standard drug therapy | A change in LVESV or LVEDV | RCT | HFrEF | NA | 1 years |  |
| Nassif et al^13^  (DEFINE-HF) | 2021 | 162 | 162 | 69 | 71 | Dapagliflozin | Standard drug therapy | Serious adverse events | RCT | HFmrEF/HFpEF | NA | 12 weeks |  |
| Solomon et al^14^  (DELIVER) | 2022 | 3131 | 3132 | 71.8 | 71.5 | Dapagliflozin | Standard drug therapy | HHF; an urgent visit for HF | RCT | HFmrEF/HFpEF | NA | 2.3 years |  |
| Anker et al^8^  (EMPEROR-Preserved) | 2021 | 2997 | 2991 | 71.8 | 71.9 | Empagliflozin | Standard drug therapy | Cardiovascular death, HHF | RCT | HFmrEF/HFpEF | NA | 26.2 months |  |
| SAVARESE et al^15^  (EMPA-REG OUTCOME) | 2021 | 4675 | 2326 | NR | NR | Empagliflozin | Standard drug therapy | Cardiovascular death, HHF | RCT | HFmrEF/HFpEF | NA | 3.1 years |  |
| Mordi et al^17^  (RECEDE-CHF) | 2020 | 12 | 11 | 69.8 | 69.8 | Empagliflozin | Standard drug therapy | Major adverse cardiac events | RCT | HFrEF | NA | 6 weeks |  |
| Omar et al^18^ | 2021 | 95 | 95 | 65 | 63 | Empagliflozin | Standard drug therapy | Major adverse cardiac events | RCT | HFrEF | NA | 12 weeks |  |
| Polito et al^19^ | 2020 | 90 | 90 | 66.1 | 67 | Sacubitril/valsartan | ACEI/  ARB | Major adverse cardiac events | RCT | HFrEF | NA | 12 months |  |
| Greene et al^20^ | 2021 | 1551 | 7857 | 77 | 78 | Sacubitril/valsartan | ACEI/  ARB | All-cause mortality; HHF | Retrospective study | HFrEF | High | 12 months |  |
| Chen et al^21^ | 2021 | 384 | 3352 | 64.9 | 66.5 | Sacubitril/valsartan | ACEI/  ARB | All-cause death,  HHF | Retrospective study | HFrEF | High | 3.6 years |  |
| Rattanavipanon et al^22^ | 2021 | 87 | 100 | 63.3 | 59.1 | Sacubitril/valsartan | ACEI/  ARB | major adverse cardiac events | Retrospective study | HFrEF | High | 12 months |  |
| Mirić et al^23^ | 2020 | 34 | 34 | 64 | 65 | Sacubitril/valsartan | ACEI/  ARB | Major adverse cardiac events | RCT | HFrEF | NA | 3 months |  |
| Gao et al^24^ | 2019 | 60 | 60 | 70.5 | 70 | Sacubitril/valsartan | ACEI/  ARB | Major adverse cardiac events | RCT | HFrEF | NA | 8 weeks |  |
| Solomon et al^25^  (PARAMOUNT) | 2019 | 2407 | 2389 | 72.7 | 72.8 | Sacubitril/valsartan | ACEI/  ARB | HHF; Death from cardiovascular causes | RCT | HFmrEF/HFpEF | NA | 8 months |  |
| Hsieh et al^26^ | 2021 | 137 | 137 | 72.7 | 72.7 | Sacubitril/valsartan | ACEI/  ARB | HHF; Death from cardiovascular causes | RCT | HFrEF | NA | 18 months |  |
| Chang et al^27^ | 2020 | 502 | 489 | 62.1 | 62.6 | Sacubitril/valsartan | ACEI/  ARB | HHF; cardiovascular death | Retrospective study | HFrEF | High | 12 months |  |
| He et al^28^ | 2021 | 251 | 251 | 62.2 | 68.6 | Sacubitril/valsartan | ACEI/  ARB | HHF; cardiovascular death | RCT | HFrEF | NA | 3 months |  |
| Damman et al^29^  (PARADIGM-HF) | 2018 | 4187 | 4212 | NR | NR | Sacubitril/valsartan | Enalapril | Composite of death from cardiovascular causes; HHF | RCT | HFrEF | NA | 8 months |  |
| Li et al^30^ | 2021 | 40 | 40 | 63.2 | 62.8 | Sacubitril/valsartan | Perindopril tert-butylamine | Major adverse cardiac events | RCT | HFrEF | NA | 12 weeks |  |
| Solomon et al^31^  (PARAMOUNT) | 2012 | 149 | 152 | 70.9 | 71.2 | Sacubitril/valsartan | Valsartan | Major adverse cardiac events | RCT | HFmrEF/HFpEF | NA | 36 weeks |  |
| Si et al^43^ | 2023 | 50 | 50 | NR | NR | Sacubitril/valsartan | ACEI/  ARB | Major adverse cardiac events | RCT | HFrEF | NA | 90 days |  |
| Zhang et al^44^ | 2023 | 43 | 43 | 69.2 | 69.9 | Sacubitril/valsartan | Standard drug therapy | Major adverse cardiac events | RCT | HFrEF | NA | 30 days |  |
| Velázquez et al^32^  （PIONEER-HF） | 2019 | 440 | 441 | 61 | 63 | Sacubitril/valsartan | Enalapril | Major adverse cardiac events | RCT | HFrEF | NA | 8 weeks |  |
| Piepoli et al^33^ | 2021 | 309 | 310 | 67.1 | 66.6 | Sacubitril/valsartan | Enalapril | Major adverse cardiac events | RCT | HFrEF | NA | 12 weeks |  |
| Riaz et al^34^ | 2021 | 1088 | 2839 | 61.5 | 62.2 | Sacubitril/valsartan | ARA | HHF; Cardiovascular death | Retrospective study | HFrEF | High | 3 years |  |
| Vaduganatan et al^9^ | 2023 | 2640 | 2622 | NR | NR | Sacubitril/valsartan | Valsartan | Worsening HF  CV death | RCT | HFmrEF/HFpEF | NA | 2.8 years |  |
| Pieske et al^40^ | 2021 | 1281 | 1285 | 72.9 | 72.4 | Sacubitril/valsartan | Standard drug therapy | Change in plasma NT-proBNP | RCT | HFmrEF/HFpEF | NA | 24 weeks |  |
| Mentz et al^41^  (PARAGLIDE-HF) | 2023 | 233 | 233 | 71.0 | 72.0 | Sacubitril/valsartan | Valsartan | Change in plasma NT-proBNP | RCT | HFmrEF/HFpEF | NA | 8 weeks |  |
| Teerlink et al^6^  （GALACTIC-HF） | 2020 | 4120 | 4112 | 64.5 | 64.5 | Omecamtiv Mecarbil | Standard drug therapy | Composite of a HF event or cardiovascular death | RCT | HFrEF | NA | 21.8 months |  |
| Lewis et al^35^ | 2022 | 185 | 91 | 63 | 67 | Omecamtiv Mecarbil | Standard drug therapy | Major adverse cardiac events | RCT | HFrEF | NA | 20 weeks |  |
| Teerlink et al^36^  （COSMIC-HF） | 2016 | 299 | 149 | 63 | 64 | Omecamtiv Mecarbil | Standard drug therapy | Major adverse cardiac events | RCT | HFrEF | NA | 20 weeks |  |
| Teerlink et al^37^  （ATOMIC-AHF） | 2016 | 303 | 303 | 66 | 66 | Omecamtiv Mecarbil | Standard drug therapy | HHF; cardiovascular death | RCT | HFrEF | NA | 30 days |  |
| Armstrong et al^7^  (VICTORIA) | 2020 | 2526 | 2524 | 67.5 | 67.2 | Vericiguat | Standard drug therapy | Composite of death from cardiovascular causes; HHF | RCT | HFrEF | NA | 10.8 months |  |
| Gheorghiade et al^38^ | 2015 | 364 | 92 | 68 | 67 | Vericiguat | Standard drug therapy | Major adverse cardiac events | RCT | HFrEF | NA | 12 weeks |  |
| Armstrong,et al^39^ | 2020 | 527 | 263 | 72 | 72.8 | Vericiguat | Standard drug therapy | Major adverse cardiac events | RCT | HFmrEF/HFpEF | NA | 24 weeks |  |

NA：Quality assessments for RCTs are provided separately in Supplementary Table 2.

Major Adverse Cardiac Events were defined as a composite endpoint including cardiovascular death, non-fatal myocardial infarction, and non-fatal stroke.

Standard therapy = Guideline-directed medical therapy (GDMT) contemporary to each trial; typically includes ACEI/ARB, beta-blockers, ±MRA

ACEI/ARB = Angiotensin-converting enzyme inhibitors or angiotensin receptor blockers as specified control

Enalapril/Valsartan/etc = Specific RAAS inhibitor with dosing as reported

Supplementary Table 2 Quality assessment of included RCTs

| Study | Random sequence generation | Allocation concealment | Blinding of participants and personnel | Blinding of outcome assessment | Incomplete outcome data | Selective reporting | Other bias | Summary  Risk |
| --- | --- | --- | --- | --- | --- | --- | --- | --- |
| McMurray et al^10^ | Low risk | Low risk | Low risk | Low risk | Low risk | Low risk | Low risk | Low |
| Nassif et al^11^ | Unclear | Unclear | Low risk | Low risk | Low risk | Low risk | Low risk | Moderate |
| Kosiborod et al^42^ | Low risk | Low risk | Low risk | Low risk | Low risk | Low risk | Low risk | Low |
| Packer et al^4^ | Low risk | Low risk | Low risk | Low risk | Low risk | Low risk | Low risk | Low |
| Singh et al^12^ | Unclear | Low risk | Low risk | Low risk | Low risk | Low risk | Low risk | Moderate |
| Nassif et al^13^ | Unclear | Low risk | Low risk | Low risk | Low risk | Low risk | Low risk | Moderate |
| Solomon et al^14^ | Low risk | Low risk | Low risk | Low risk | Low risk | Low risk | Low risk | Low |
| Anker et al^8^ | Low risk | Low risk | Low risk | Low risk | Low risk | Low risk | Low risk | Low |
| Savarese et al^15^ | Low risk | Low risk | Low risk | Low risk | Low risk | Low risk | Low risk | Low |
| Mordi et al^17^ | Unclear | Low risk | Low risk | Low risk | Low risk | Low risk | Low risk | Moderate |
| Omar et al^18^ | Low risk | Low risk | Low risk | Low risk | Low risk | Low risk | Low risk | Low |
| Polito et al^19^ | Unclear | Unclear | Unclear | Unclear | Low risk | Low risk | Low risk | High |
| Mirić et al^23^ | Unclear | High risk | High risk | Low risk | Low risk | Low risk | Low risk | High |
| Gao et al^24^ | Unclear | High risk | High risk | Low risk | Low risk | Low risk | Low risk | High |
| Solomon et al^25^ | Low risk | Low risk | Low risk | Low risk | Low risk | Low risk | Low risk | Low |
| Hsieh et al^26^ | Unclear | High risk | High risk | Low risk | Low risk | Low risk | Low risk | High |
| He et al^28^ | High risk | High risk | High risk | Low risk | Low risk | Low risk | Low risk | High |
| Damman et al^29^ | Low risk | Low risk | Low risk | Low risk | Low risk | Low risk | Low risk | Low |
| Li et al^30^ | High risk | High risk | High risk | Low risk | Low risk | Low risk | Low risk | High |
| Solomon et al^31^ | Low risk | Low risk | Low risk | Low risk | Low risk | Low risk | Low risk | Low |
| Si et al^43^ | High risk | High risk | High risk | Low risk | Low risk | Low risk | Unclear | High |
| Zhang et al^44^ | Low risk | Low risk | Unclear | Low risk | Low risk | Low risk | Low risk | Moderate |
| Velázquez et al^32^ | Low risk | Low risk | Low risk | Low risk | Low risk | Low risk | Low risk | Low |
| Vaduganatan et al^9^ | Low risk | Low risk | Low risk | Low risk | Low risk | Low risk | Low risk | Low |
| Pieske et al^40^ | Low risk | Low risk | Low risk | Low risk | Low risk | Low risk | Low risk | Low |
| Mentz et al^41^ | Low risk | Low risk | Low risk | Low risk | Low risk | Low risk | Low risk | Low |
| Piepoli et al^33^ | Low risk | Low risk | Low risk | Low risk | Low risk | Low risk | Low risk | Low |
| Teerlink et al^6^ | Low risk | Low risk | Low risk | Low risk | Low risk | Low risk | Low risk | Low |
| Lewis et al^35^ | Low risk | Low risk | Low risk | Low risk | Low risk | Low risk | Low risk | Low |
| Teerlink et al^36^ | Low risk | Low risk | Low risk | Low risk | Low risk | Low risk | Low risk | Low |
| Teerlink et al^37^ | Low risk | Low risk | Low risk | Low risk | Low risk | Low risk | Low risk | Low |
| Armstrong et al^7^ | Low risk | Low risk | Low risk | Low risk | Low risk | Low risk | Low risk | Low |
| Gheorghiade et al^38^ | Low risk | Low risk | Low risk | Low risk | Low risk | Low risk | Low risk | Low |
| Armstrong et al^39^ | Low risk | Low risk | Low risk | Low risk | Low risk | Low risk | Low risk | Low |

Low risk: adequate methods unlikely to introduce bias; High risk: inadequate methods likely to introduce bias; Unclear risk: insufficient information to judge.

Supplementary Table 3 Publication bias of cardiovascular outcomes

| **Outcomes** | **Begg** | | | **Egger** | **Number of Studies Included** |
| --- | --- | --- | --- | --- | --- |
| **SGLT2i vs Placebo** | |  | |  |  |
| Cardiovascular death | 1.00 | | | 0.40 | 7 |
| HHF | 0.23 | | | 0.35 | 7 |
| Death from any cause | 0.25 | | | 0.33 | 9 |
| Serious adverse events | 0.45 | | | 0.44 | 6 |
| Volume depletion | 1.00 | | | 0.49 | 5 |
| Major hypoglycemia | 0.46 | | | 0.39 | 6 |
| Amputation | 0.30 | | | 0.15 | 5 |
| SBP | 0.74 | | | 0.15 | 6 |
| **Sacubitril/Valsartan vs Placebo** | | |  |  |  |
| Cardiovascular death | 0.90 | | | 0.25 | 8 |
| HHF | 0.74 | | | 0.23 | 14 |
| Death from any cause | 0.10 | | | 0.19 | 14 |
| Hypotension | 1.00 | | | 0.95 | 10 |
| Angioedema | 0.46 | | | 0.07 | 8 |
| Hyperkalemia | 1.00 | | | 0.40 | 8 |

P < 0.10 indicates potential publication bias.

**Supplementary Figure 1.** Funnel plots for publication bias assessment in SGLT2i meta-analysis.
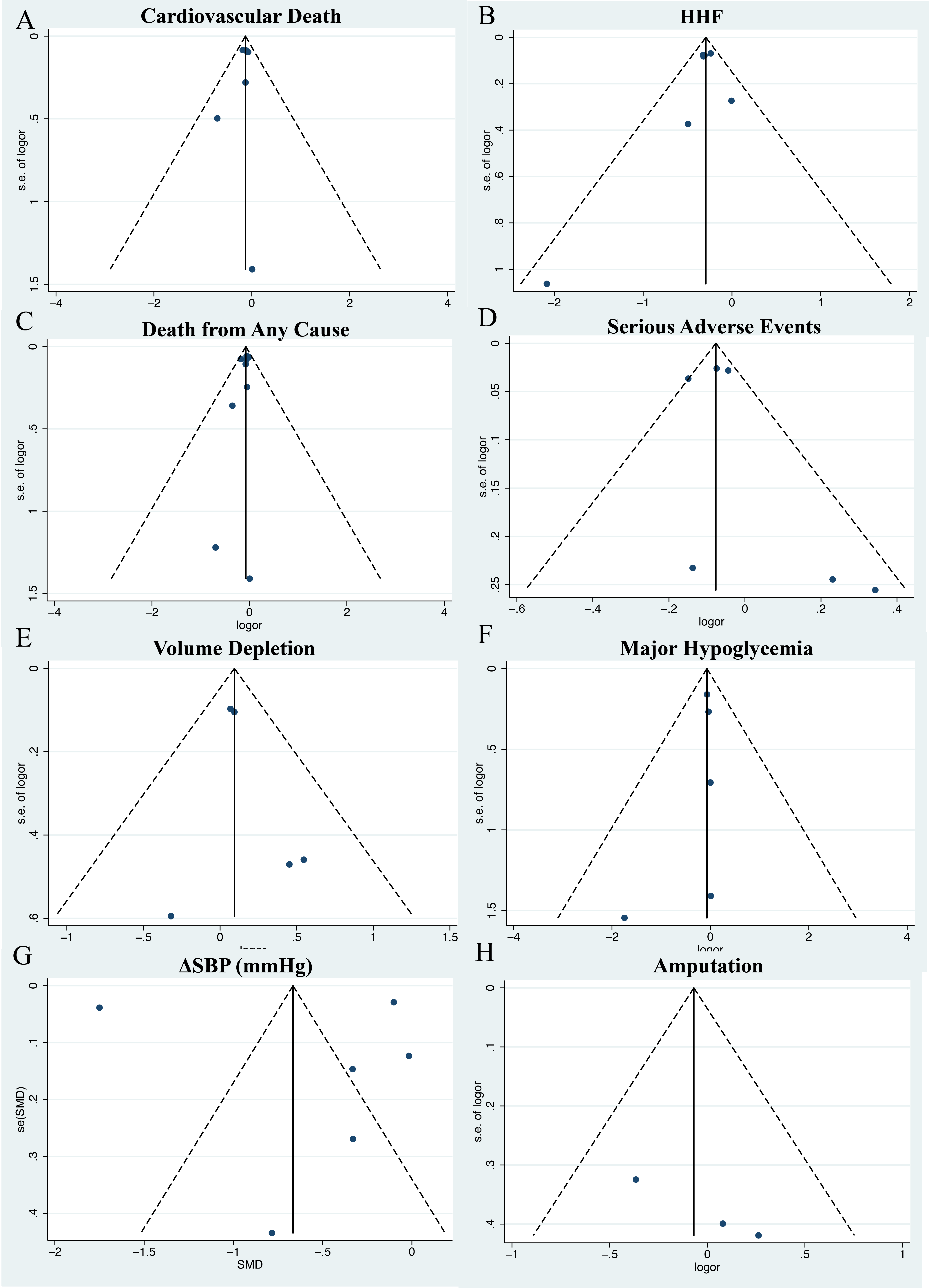


**Supplementary Figure 2.** Funnel plots for publication bias assessment in sacubitril/valsartan meta-analysis.


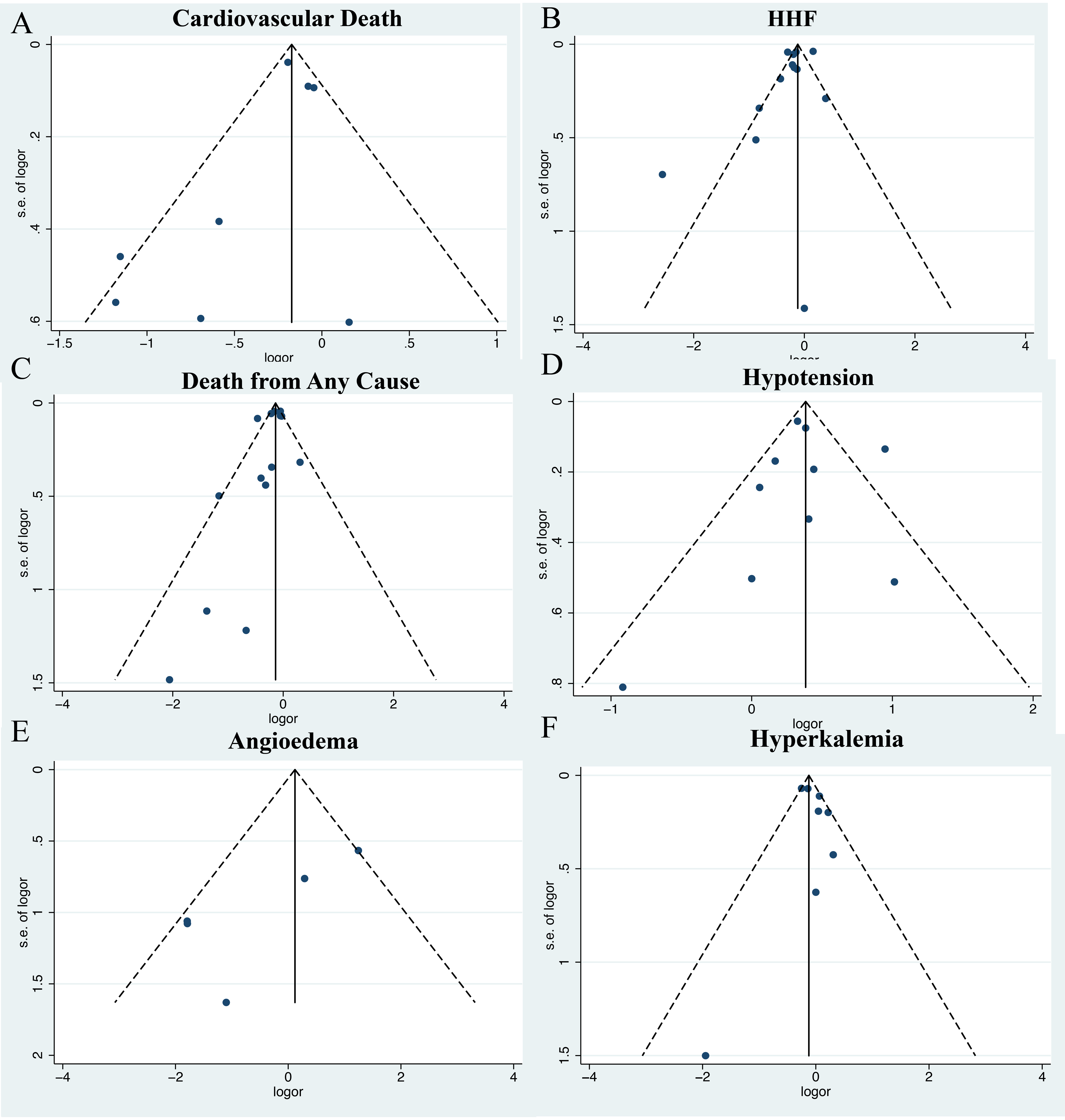


Literature Search Strategy

A comprehensive systematic literature search was conducted in five electronic databases: Web of Science, PubMed, the Cochrane Library, EMBASE, and China National Knowledge Infrastructure (CNKI), from inception to February 17, 2025, without language restrictions. The search strategy combined Medical Subject Headings (MeSH) terms with free-text keywords using Boolean operators.

Search Strategy Components:

For heart failure, we used: "heart failure"[MeSH Terms] OR "heart failure"[Title/Abstract] OR "cardiac failure"[Title/Abstract] OR "HF"[Title/Abstract] OR "HFrEF"[Title/Abstract] OR "HFpEF"[Title/Abstract] OR "HFmrEF"[Title/Abstract]

AND

For SGLT2 inhibitors, we used: "sodium-glucose transporter 2 inhibitors"[MeSH Terms] OR "SGLT2 inhibitor*"[Title/Abstract] OR "sodium glucose cotransporter 2 inhibitor*"[Title/Abstract] OR "dapagliflozin"[Title/Abstract] OR "empagliflozin"[Title/Abstract] OR "canagliflozin"[Title/Abstract] OR "sotagliflozin"[Title/Abstract]

OR

For sacubitril/valsartan, we used: "sacubitril and valsartan drug combination"[MeSH Terms] OR "sacubitril/valsartan"[Title/Abstract] OR "LCZ696"[Title/Abstract] OR "Entresto"[Title/Abstract]

OR

For vericiguat, we used: "vericiguat"[Title/Abstract] OR "Verquvo"[Title/Abstract] OR "BAY 1021189"[Title/Abstract]

OR

For omecamtiv mecarbil, we used: "omecamtiv mecarbil"[Title/Abstract] OR "CK-1827452"[Title/Abstract]
